# THE NET ON THE RIVER WHERE VERY LITTLE HAS BEEN CAUGHT: DIFFERENCES IN MEMORY FOR DISCOURSE IN YOUNGER AND OLDER ADULTS

**DOI:** 10.1093/geroni/igad104.2608

**Published:** 2023-12-21

**Authors:** Marjorie Getz

**Affiliations:** Methodist College, Peoria, Illinois, United States

## Abstract

Experiential learning is key for courses in a gerontology certificate program at a midwestern nursing and health science college. One curricular emphasis is the uniqueness of every older adult that the student will encounter as they move from a novice student role to professional role mastery several years into their careers. To assist in this transition, one course has a unit on memory for discourse, especially memory for printed materials—essential in support of understanding medical conditions and treatment follow-through. Our project had college students compare their memory for short stories with memories of “students” from an OLLI Program who attended this class. The project, in an adult development course, provides students with community service, direct contact with older adults and reflective practices to integrate course content with this activity. Qualitative analyses were used. Content analyses of reflective essays identified four themes (a) older adult focus on key story propositions as well as incidental propositions with tie-in to acquired knowledge on attention/memory from course work (b) frequent use of situational schema based on greater world knowledge held by older adults and implications of this type of discourse in clinical settings (c) differences, that favored older adults, between groups in memory for details in structured versus unstructured stories, and (d) differences in style of recall for stories between age groups. Reflections were encouraged to tie new age-related knowledge about memory processes into better client follow through. Students also reflected on ways to make memory more optimal in clinical settings.

